# Anatomical, histological, and histochemical alterations in portio vaginalis uteri with an evaluation of the vaginal artery vascularity during the luteal and early pregnant stages in domestic buffaloes (Bubalus bubalis)

**DOI:** 10.1186/s12917-023-03816-9

**Published:** 2023-12-06

**Authors:** Yara S. Abouelela, Nora A. Shaker, Khaled H. El-Shahat, Dina W. Bashir, Hossam R. El-Sherbiny, Elshymaa A. Abdelnaby

**Affiliations:** 1 Anatomy and Embryology Department, Faculty of Veterinary Medicine, Cairo University, Giza Square, Giza, 12211 Egypt; 2 Theriogenology Department, Faculty of Veterinary Medicine, Cairo University, Giza, Egypt; 3 Cytology and Histology Department, Faculty of Veterinary Medicine, Cairo University, Giza, Egypt; 4 Department of Clinical Sciences, College of Veterinary Medicine, King Faisal University, Alahsa, Saudi Arabia

**Keywords:** Bovine, Doppler, Histology, Cervix, Vaginal Artery

## Abstract

The portio-vaginalis uteri (PVU) and its mucus secretion have shown an essential role in conception. A significant endeavour to improve buffaloes' reproductive efficiency is the investigation of their basic reproductive pattern, which provides a reference for applications in breeding and pregnancy. The present study aimed to evaluate the anatomical and histological alterations in PVU regarding to the vaginal artery (VA) hemodynamic at luteal and early pregnant stages in buffalos. Egyptian live buffaloes (*n* = 16) and fresh genitals (*n* = 25) of mature buffalo were used. Different luteal and early pregnant stages were macroscopically identified with the shape and mucosal colouration with discharges of the PVU. Histological examination showed a significant difference in area % of alcian blue and periodic acid Schiff positive granules which considered an indication for presence of acidic and neutral mucins respectively in the epithelial cells of PVU mucosa which increased in pregnant stage than in other luteal stages. VA assessment demonstrated an increase in luminal diameter and thickness of tunica muscularis in pregnant stage than other stages (*P* < 0.05). Middle uterine (MUA) and VA arteries peak velocity point (PSV mm/sec) were elevated (*P* < 0.05) in pregnant stage, with a marked reduction in both resistance and pulsatility indices (RI and PI), and ratio of systolic /diastolic (S/D). Positive correlation was detected between VA. PSV and, MUA. PSV (*r* = 0.87), but a negative relation was detected with VA. S/D (*r* = -0.77), VA.PI (*r* = -0.89), VA. RI (*r* = -0.97), MUA. S/D (*r* = -0.94), MUA. PI (*r* = -0.85), and MUA. RI (*r* = -0.88). Doppler indices were negatively corrected with the VA. PSV (*r* = -0.68). It was concluded that there was a significant alterations in histological features of the cervical PVU at different physiological stages (luteal and early pregnant) in buffalos in relation to the MUA and VA hemodynamic pattern and that hypotheses can be established regarding the female cyclicity that affected by both arteries hemodynamics change.

## Introduction

The reproductive performance in buffalo is decreased due to its affection by various diseases [1, 2] as inflammation of cervix which become painful and swollen and cervical tumor that alter shape and appearance of PVU. So, studying a basic reproductive pattern is of great effort for enhancing the reproductive efficiency of buffaloes [3] .The macroscopical appearance of the portio vaginal uteri (PVU) can be found in many different shapes including slit, bud (papilla), rose, spiral, rosette, star, bunch and tuber shape varies according to the age as mentioned in the previous studies in sheep [4–6]. Otherwise, the histological features of the genital organs could be differentiated depending on their reproductive status and its seasonal difference [7]. Moreover, the cervical mucus secretion plays an essential role in conception. The properties of cervical secretion of cattle and buffaloes, as well as its quantity, vary according to the hormonal effect which corresponds to the stages of the estrus cycle in addition to ovulation time, that affect the empower or impede sperm motility [8, 9]. The luteal and early pregnancy phases are linked with marked variations in the cardiovascular hemodynamic system, for example Doppler indices reduction, raised blood flow volume, and especially vascular resistance index [10, 11].

The cervix with PVU in the non-pregnant and early pregnant females could be easily visualized by ultrasound [12, 13]. The vascular distribution, density, morphological appearance of blood vessels, and their pathological alterations can be obtained using the colour Doppler approach [14, 15] regarding the presence of the vaginal artery (VA). However, color mode of Doppler technology emerges as a practical useful tool for blood flow of small organs like ovary, vagina, and lymph node [16]. The spectral form of Doppler modes is mostly used and preferred [17–20], but this mode does not provide the sufficient data regarding the blood flow of small organs like ovary, vagina, and lymph node.

The changes in the morphology of PVU and vascular perfusion of VA of domestic buffaloes in different stages (luteal and early pregnant) were not assessed in the previous literatures and this is considered a research gap in addition knowing the vasculature of vaginal arteries at various stages is so critical. Thus, the present study aimed to investigate the anatomical, histological, and histochemical alterations of the PVU of the domestic buffalo regarding their vascular supply of the vaginal artery which gives a guide used in breeding and pregnancy applications.

## Materials and methods

### Ethical approval

All experimental protocols were approved by the institutional animal use committee (IACUC) of Veterinary Medicine at Cairo University with a certified licensee number (Vet CU12/10/2021/387)

### Animal selection, feeding and housing

The present work was applied on 16 live cyclic pluriparous adult Egyptian domestic buffalo with an age 9-14 years for Doppler examination. These animals were kept in the large animal farm at the theriogenology department Faculty of Veterinary Medicine, Cairo University (30.0276◦N, 31.2101◦E). They were fed a mixed ration (40% concentrate, 60%forage, and dry matter) with free access of water. All females underwent a routine weekly assessment (every week for three successive estrous cycles) to check the genital tract functionality and exclude any gynecological and cardiovascular problems using an ultrasound device (EXAGO–made in France) adjusted with a linear probe with a frequency 6-10 MHz [21, 22]. Buffalos were synchronized using Ovsynch protocol that was previously done using gonadotropin, prostaglandins, and another dose of gonadotropin (GPG) [11]. All buffalos were examined after synchronization (*n* =16), the first estrous cycle after the synchronization was examined. Some of the examined animals were served as a group I (Group I; *n* = 6) enter in luteal stages to be evaluated. The other ten buffalos were selected based on their genetic character to be pregnant once mated naturally by an excellent bull after the second gonadotropin injection in the Ovsynch program by 21 hours [23]. All mated buffalos were examined for pregnancy at day 30, only 6 became pregnant and those were examined after that for uterine and vaginal blood flow were considered as group II (Group II; *n* = 6). The ovulation was confirmed by the ultrasonographic evaluation of the preovulatory follicles disappearance [24].

### Samples collection

**Fig. 1:**
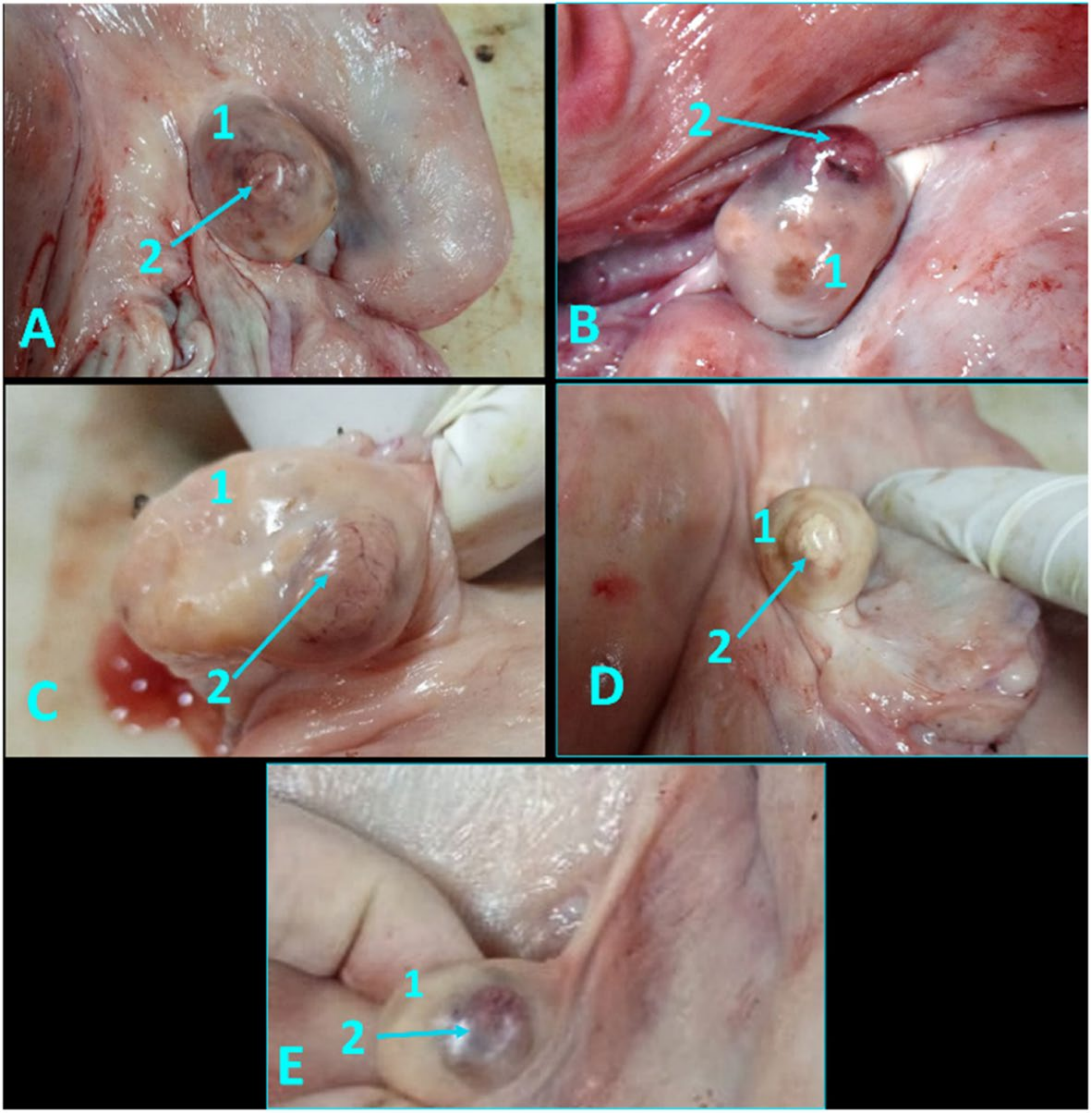
Corpus luteum in the buffalo ovary at different luteal stages as **A** Represented an early luteal (stage I, 1–5 days), **B** Demonstrated a mid-luteal (stage II, 6–10 days), **C** Showed a mid-luteal (stage III, 11–16 days), **D** Showed a late luteal (stage IV, 17–20 days), and **E** Demonstrated corpus luteum gravidities at early stage of pregnancy. N.B: 1 = ovary, and 2 = corpus luteum

Fresh female genitals of mature buffalo obtained from great Cairo abattoirs in Egypt over six months for anatomical and histological examination (*n *= 25). All specimens were inspected within two hours of sacrification at Faculty of veterinary medicine, Cairo University. The twenty-five specimens were subdivided into twenty for anatomical and histological studies and the other five specimens for vascular anatomical architecture of vaginal artery (VA). The twenty genital specimens were categorized into early pregnant genitalia (*n *= 4), non-pregnant genitalia (*n *= 16) and the non-pregnant genitalia were divided according to the size, consistency, and shape of the corpus luteum inspected on the ovary into different luteal stages: early luteal (Stage I, *n *= 4) at 1–5 days that CL appeared small, reddish, and soft in ovarian surface (Fig. 1A); mid luteal (Stage II, *n *= 4 from 6–10 days which ovary had a large, brownish, and harder CL (Fig. 1B); and Stage III, *n *= 4 at 11–16 days as CL appeared fibrous, pale, and hard (Fig. 1C); and late luteal stage (Stage IV, *n *= 4), from17–20 days CL be seen fibrous, yellowish and hard (Fig. 1D). While, in early pregnant stage, (*n *= 4) CL became light reddish coloration with enlargement of the uterine horn with presence of foetus (Fig. 1E). This classification was defined depends on Daghash, et al.; Baithalu et al., [11, 25].

### Anatomical inspection

Specimens were examined in a fresh state to recognize the shape and mucosal colouration of PVU along with its mucoid discharges. The width of PVU was measured by using digital callipers. The other five buffalo's genitalia specimens, for studying the vaginal arteries were cannulated to clear the blood clot by thoroughly washing with normal saline then injected by coloured red emulsion of 60% gum milk latex using ROTRING ink. Specimens were frozen for 48 hours before dissection**.** Photos were snapped by a digital camera and influenced by Photoshop ccx64 version.

### Histological examination

After the anatomical examination of the same twenty genitalia specimens at the different luteal and early pregnant stages, their PVU and vaginal arteries were carefully removed, dissected out, and immediately fixed in 10% neutral buffered formalin for 48 hours. They were cleaned, trimmed, and dehydrated in ascending alcohol concentrations before being cleaned with xylene and fixed in paraffin wax. Using a rotatory microtome, 4 μm sections were cut. Then they were dewaxed and stained with Hematoxylin and eosin (H&E) for general tissue structure and histochemical stains; alcian blue (AB) pH 2.5 and periodic acid Schiff (PAS) for demonstration of acidic and neutral mucins respectively in the portio vaginalis specimens. In addition to, orcein stain for the vaginal artery. These samples were then examined under a light microscope [26].

#### Evaluation of histochemical observations “area percentage”

Histochemically stained AB and PAS sections were analyzed using a Leica Quin 500 analyzer computer system (Leica Microsystems, Switzerland). The image analyzer was automatically calibrated in order to convert the measurement units (pixels) produced by the image analyzer programme into actual micrometre units. The histochemical stain was assessed using light microscopy and a magnification of X400 in 5 fields from several slides at each stage to determine its percentage of an area within a standard measurement frame. Regardless of the staining intensity, the locations exhibiting the positive histochemical stain reaction were picked for assessment. A blue binary color was used to mask these places so that the computer system could measure them. Each specimen's mean value and standard error (SE) were calculated, and the results were statistically analyzed.

#### Histomorphometrical analysis of vaginal artery

Five cross-sections from the vaginal artery in each luteal and early pregnant stages were measured by an X40 eyepiece. To determine the histomorphometric parameters “the luminal diameter and the thickness of the tunica muscularis” using statistical analysis, a computerized microscopic image analyzer attached for the full high-definition microscopic camera (Leica Microsystems, Germany) was used.

### Identification of the uterus, uterine blood flow, vagina, and vaginal blood flow

The urinary bladder considered as a landmark for ultrasonography due to urine content to find the cervical rings, then the uterus with two uterine horns. Measurement of uterine horns diameters (right and left; mm) and endometrial thickness (mm) using a rectal probe in a grey b-mode image was performed. The endometrial thickness was identified from the two echogenic borders with a white line present inside. The color Doppler mode was activated to detect the uterine and vaginal artery’s locations and blood flow with the device setting as follows (pulse repetition frequency was 3.5 kHz, the Doppler gate size was 0.5 mm, and the pass filter was set at 4 Hz. The Middle uterine artery (MUA) was estimated close to the internal iliac artery near the uterocervical junction [11, 27, 28].

The anatomical location of the PVU was determined by the rectal assessment after removing the faecal matter starting with b-mode and then Doppler was activated with an angle of insonation less than 60° [29]. Moreover, based on the anatomical position, the vaginal artery spectral graphs were calculated along different time points at early, mid, late luteal and early pregnant stages. The following parameters were determined from each frozen pulsed wave Doppler image of the known artery: peak point of velocity due to maximum contraction (PSV; mm/sec), the endpoint of velocity due to maximum relaxation (EDV; mm/sec), the important ratio between systolic /diastolic (S/D), and two Doppler indices expressed by resistance and pulsatility index (RI and PI) as previously measured in the same animals [11].

### Statistical analysis

Data were presented as mean and standard error. The significance of the means was determined by one way analysis of variance (ANOVA) using SPSS version 28 software. They were confirmed by the least significant difference LSD post hoc test. Significant differences are those with a *P* value < 0.05.

## Results

### Anatomical and histological observations of the PVU

**Fig. 2:**
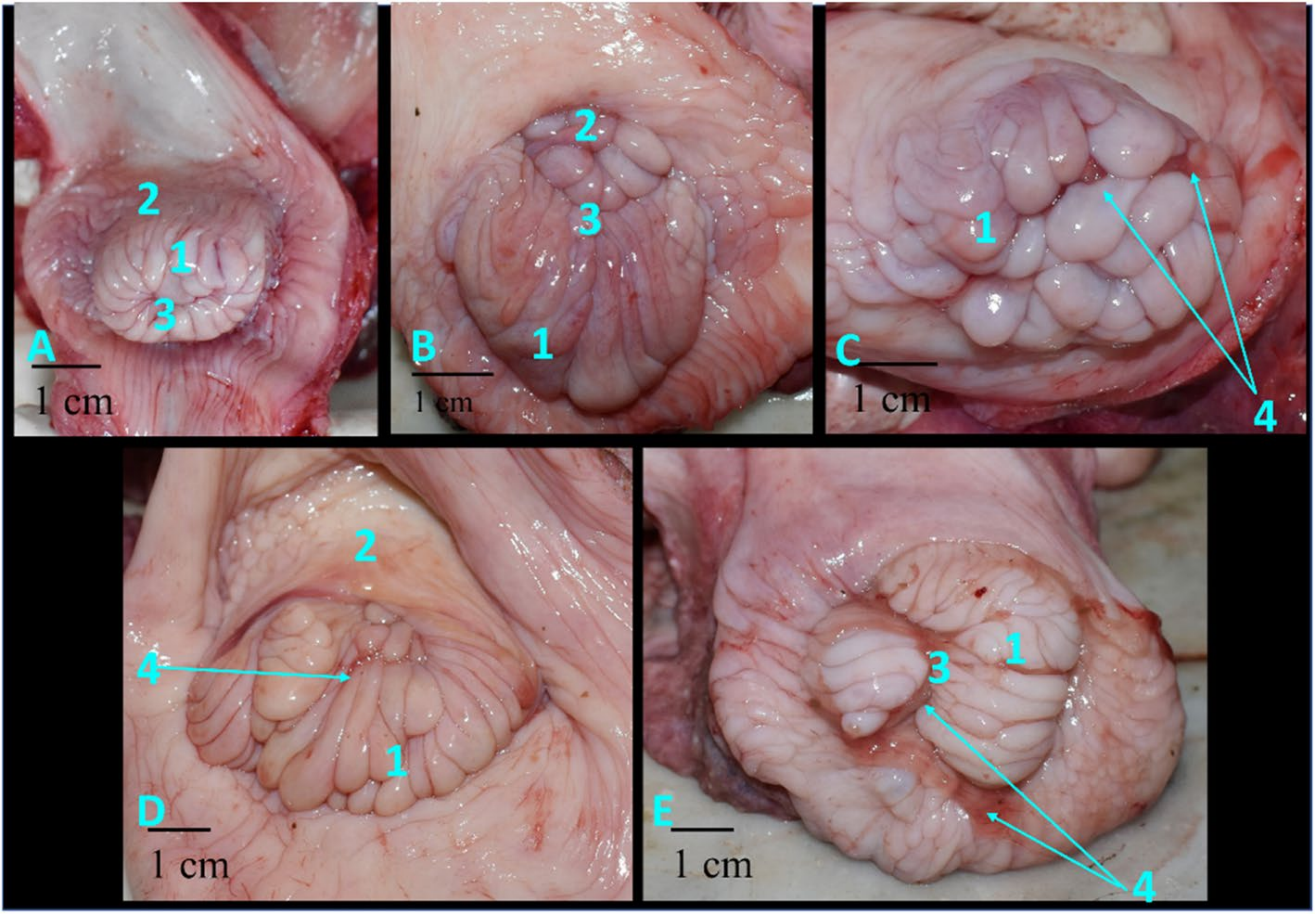
Portio vaginalis uteri in different luteal stages in Egyptian buffalo. **A** Early luteal, **B** Mid-luteal (stage II), **C** Mid-luteal (stage III), **D** Late luteal, **E** Pregnant 1- foliae of protio, 2- fornix, 3- uterine orifice, 4- mucoid secretion

There were three different luteal stages according to the size and shape of the corpus luteum inspected on the ovary: early luteal, mid-luteal (stages II-III), late-luteal, in addition to the early pregnant stage. Early luteal (stage I), the PVU was small protruded contracted mass had several cervical foliae that were recognized as papilla shape with the same size, length, and diameter. The total width was 25 mm. External uterine orifices located centrally (Fig. 2A). Though in the mid-luteal (stage II), the cervical foliae that located ventrally was increased in length and diameter and prolapsed caudally as a compact mass. While the dorsal part of foliae was small in size. The total width was 30mm. the external uterine orifice becomes dorsally (Fig. 2B).In the mid-luteal (stage III), the PVU were circular rosette in shape. Cervical foliae was swollen and prolapsed in equal size (length and diameter) in a regular manner and become flab. The total width was 40mm. The external uterine orifice became dorsally with few amounts of watery clear vaginal secretion (Fig. 2C).While in late luteal (stage IV), the cervical foliae became more flaccid rosette with increasing in diameter, length, thickness, and distributed ventrally in an irregular manner. The total width was 55 mm as well as the external uterine orifices placed dorsally and covered with watery clear vaginal secretion (Fig. 2D).

Even as in the early pregnant stage, the cervical foliae become contracted and rearranged with an increase in size and diameter. The total width was 40mm and there was thick abundant vaginal mucoid secretion covered the PVU and closed the external uterine orifice (Fig. 2E).

**Fig. 3:**
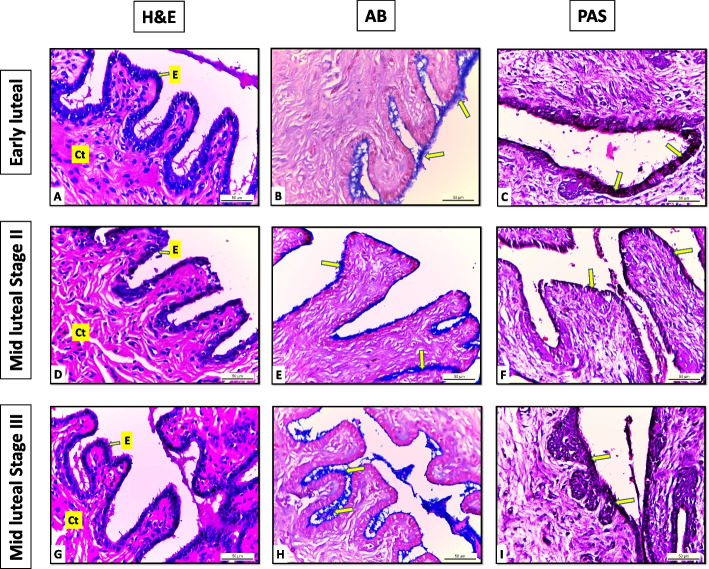
Sections of portio vaginalis uteri of Egyptian buffalo at different luteal stages A:C early luteal, D:F mid-luteal (stage II), G:I mid-luteal (stage III) E: Epithelium “simple columnar mucous secreting”, Ct: Connective tissue “fibro elastic propria submucosa”, Arrows: indicate positive reaction for AB & PAS stains “few in the early and mid-luteal (stage II) and moderate in the mid-luteal (stage III). X400

**Fig. 4:**
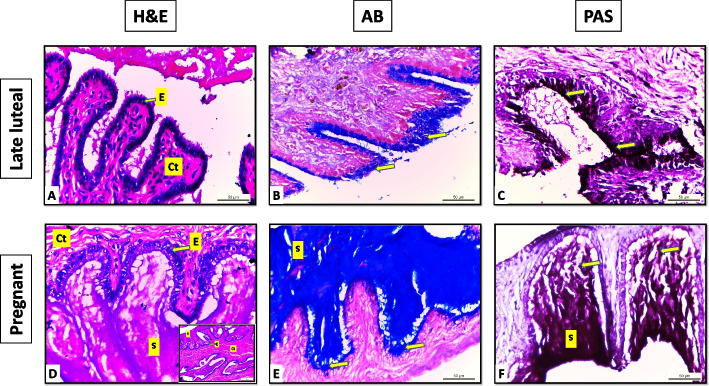
Sections of portio vaginalis uteri of Egyptian buffalo at different luteal stages A:C late luteal, D:F pregnant, E: Epithelium “simple columnar mucous secreting”, Ct: Connective tissue “fibro elastic propria submucosa”, S: Secretion, Arrows: indicate positive reaction for AB & PAS stains “slightly abundant in the late luteal stage and more abundant in the pregnant stage” X400, inside cube X 100

Light microscopic examination of H&E-stained sections of PVU of Egyptian buffalo at different luteal stages showed that the tunica mucosa was composed of folds lined by mucous secreting columnar cells “lamina epithelialis” rest on fibroelastic connective tissue propria- submucosa (Figs. 3 & 4).

Examination of the histochemical stains (AB & PAS) of the same sections demonstrated a positive reaction in the epithelial cell's apical cytoplasm increased gradually in all luteal stages till become more abundant in the pregnant stage. The reaction was few in the early and mid-luteal stage II and moderate in the mid-luteal stage III (Fig. 3). Meanwhile, in the late luteal stage, it becomes slightly abundant and more abundant in the pregnant stage (Fig. 4).

Analysis of the data showed non-significant differences in the area % covered by AB positive reaction in the early and mid-luteal stage II (18.7 ± 1 and 29.5 ± 1). However, significant differences were recorded between other stages (43.3 ± 3, 58.2 ± 2, and 103.4 ± 6) mid-luteal stage III, late luteal, and pregnant stages respectively. On the other hand, the area % of PAS-positive reaction showed a significant difference in all stages (41.2 ± 2, 59.02 ± 2, 69.2 ± 1, 89.04 ± 2, and 109 ± 3) early stage I, mid-luteal stage II, III, late luteal, and pregnant stages respectively (Table 1).

**Table 1:** Alcian blue and periodic acid Schiff area percentage of portio vaginalis uteri in Egyptian buffalo at different luteal and pregnant stages

Means ± SE	Stages				
	Early luteal	Mid luteal “stage II”	Mid luteal “stage III”	Late luteal	Pregnant
**AB Area**%	18.7 ± 1^a^	29.5 ± 1^a^	43.3 ± 3^b^	58.2 ± 2^c^	103.4 ± 6^d^
**PAS Area %**	41.2 ± 2^a^	59.02 ± 2^b^	69.2 ± 1^c^	89.04 ± 2^d^	109 ± 3^e^

^a,b,c,d,e^ Mean values with different superscripts in the same row indicate significant difference (*P* ≤ 0.05)

### The vaginal artery examination

**Fig. 5:**
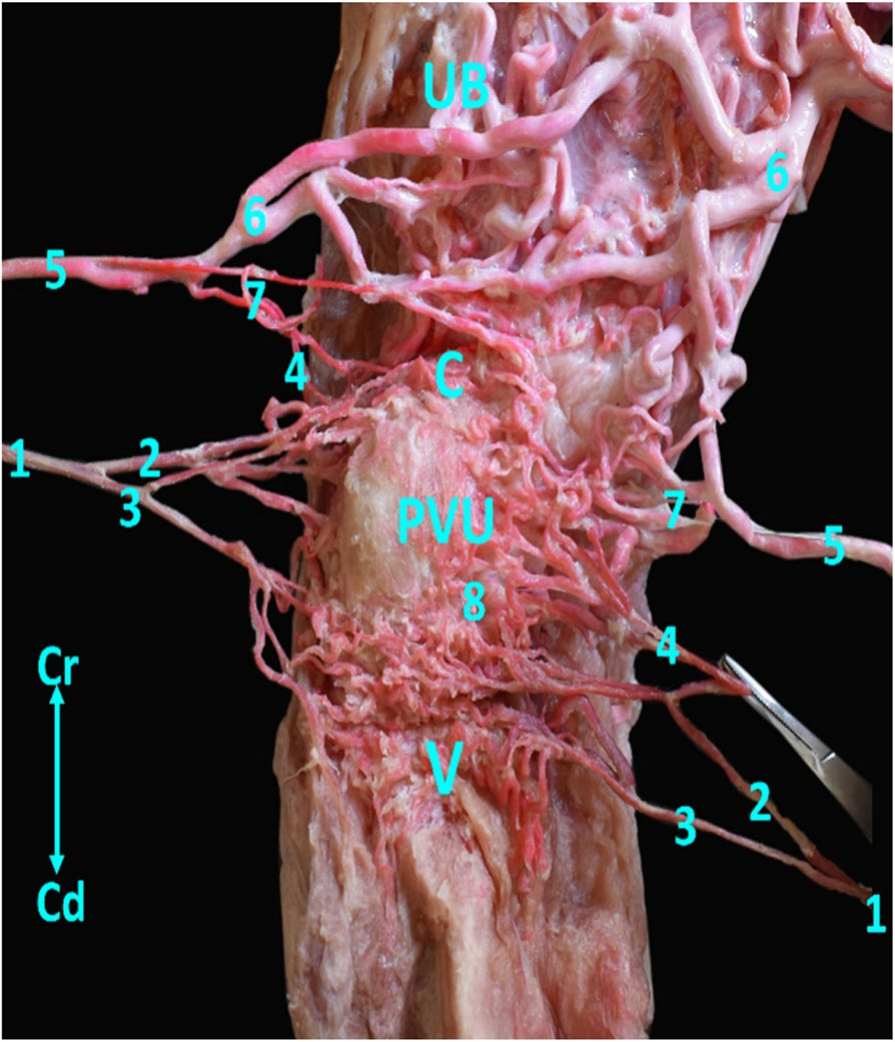
Distribution of the vaginal artery in Egyptian buffalo. UB = uterine body, C = cervix, PVU = portio vaginalis uteri, V = vagina. 1- vaginal artery, 2-cranial br of vaginal artery, 3- caudal br of vaginal artery, 4- communicating br with uterine artery, 5- ventral br of middle uterine artery, 6- Lateral br of ventral middle uterine artery, 7- Medial br of ventral middle uterine artery, 8- small, ramified twigs

The vaginal artery (Fig. 5) originated from the internal iliac artery at the level of the 6^th^ lumbar vertebrae. It is divided into a cranial large branch (Fig. 5) and a caudal small one (Fig. 5). The cranial branch supplied the cervix through three twigs which reinforced with the last secondary branches of the ventral branch of the middle uterine artery (Fig. 5), while the caudal artery divided into 2-3 twigs terminated at the cranial part of the vagina to supply the cranial part of vagina and PVU.

There were no significant changes in all examined parameters including anterior uteri horn diameter (H; mm), endometrial thickness (ET; mm), middle uterine artery diameter (MUA; mm), and uterine hemodynamic parameters (peak and end points of velocities [PSV, EDV mm/sec, and systolic over diastolic ratio S/D], pulsatility index [PI], and resistance index [RI] of the MUA) between the two sides (right and left) of the uterine horn (Table 2).

**Table 2:** The difference in the left and right side in the uterine horn, endometrial thickness, and middle uterine artery morphometry and hemodynamic in Egyptian domestic buffalos at luteal stage after ovulation. Data are expressed as means ± standard error

Parameter (Mean ± SE)	Left side	Right side	*P*-Value
H diameter (mm)	11.02 ± 0.25	10.84 ± 0.44	0.84
ET (mm)	13.65 ± 3.26	13.48 ± 1.25	0.95
MUA diameter (mm)	11.25 ± 0.22	11.63 ± 0.52	0.55
Middle uterine artery vascularization			
MUA. PSV (mm/s)	122.21 ± 22.36	126.25 ± 21.65	0.45
MUA. EDV (mm/s)	35.62 ± 5.66	38.65 ± 3.25	0.32
MUA. S/D	3.55 ± 0.25	3.65 ± 0.32	0.85
MUA. PI	1.55 ± 0.02	1.53 ± 0.02	0.95
MUA. RI	0.79 ± 0.02	0.81 ± 0.01	0.71

There was no significant differences between right and left side of the genital tract during luteal phase

*SD* Standard error, *H* Horn, *ET* Endometrium thickness, *MUA* Middle uterine artery, *PSV* Peak point of velocity, *EDV* End point of velocity, *S/D* Systolic over diastolic ratio, *PI* Pulsatility Doppler index, and *RI* Resistance Doppler index

Uterine horn diameter (H; mm), endometrial thickness (ET; mm), vaginal artery diameter (VA; mm), and vaginal folds thickness (mm) were increased (*P *= 0.01) in early pregnant stage compared to luteal stages. In addition, both vaginal and middle uterine arteries maximum velocity expressed by peak systolic and (PSV; mm /sec) were increased (*P *= 0.02) in early pregnant stage compared to luteal stages. Finally, VA.PI and RI with MUA PI and RI were significantly decreased (*P *= 0.01) in early pregnant stage compared to luteal stages, with no significant changes detected in VAS/D, MUA S/D, VA end velocity (EDV; mm/sec), and MUA EDV (Table 3).

**Table 3:** The differences in the uterine horn diameter, endometrial thickness, vaginal folds thickness, vaginal artery morphometry, uterine artery morphometry and hemodynamics in Egyptian domestic buffalos at luteal and early pregnant stages. Data are expressed as means ± standard error

Parameter (Mean ± SE)	Diestrous phase				Early pregnant stage
	**Early luteal** **“stage I”**	**Mid luteal “stage II”**	**Mid luteal “stage III”**	**Late luteal “stage IV”**	
**H diameter (mm)**	11.52 ± 1.54^a^	11.62 ± 0.74^a^	11.66 ± 0.22^a^	11.64 ± 0.33^a^	12.87 ± 0.33^b^
**ET (mm)**	12.98 ± 0.65^a^	13.04 ± 0.45^a^	13.08 ± 0.62^a^	13.15 ± 0.25^a^	16.55 ± 0.25^b^
**Vaginal folds thickness (mm)**	10.02 ± 0.55^a^	10.13 ± 0.33^a^	10.54 ± 0.22^ab^	10.85 ± 0.78^ab^	11.65 ± 1.02^b^
**VA diameter (mm)**	11.01 ± 0.12^a^	11.01 = 5 ± 0.21^a^	11.62 ± 0.41^ab^	11.85 ± 0.66^ab^	13.89 ± 0.85^b^
**Middle uterine artery vascularization**					
**MUA. PSV (mm/s)**	122.21 ± 22.36^a^	123.55 ± 21.35^a^	128.32 ± 22.66^ab^	129.62 ± 26.58^ab^	133.25 ± 33.25^b^
**MUA. EDV (mm/s)**	35.62 ± 5.66	35.66 ± 2.32	35.69 ± 1.25	36.25 ± 2.31	35.95 ± 0.62
**MUA. S/D**	3.55 ± 0.25	3.62 ± 0.32	3.55 ± 0.22	3.64 ± 0.14	3.72 ± 0.52
**MUA. PI**	1.55 ± 0.02^b^	1.54 ± 0.01^b^	1.49 ± 0.01^ab^	1.49 ± 0.01^ab^	1.21 ± 0.02^a^
**MUA. RI**	0.79 ± 0.02^b^	0.75 ± 0.01^b^	0.75 ± 0.01^b^	0.68 ± 0.01^ab^	0.51 ± 0.02^a^
**Vaginal artery vascularization**					
**VA. PSV (mm/s)**	118.51 ± 33.25^a^	121.32 ± 34.27^a^	126.66 ± 21.33^ab^	127.25 ± 12.32^ab^	132.66 ± 15.66^b^
**VA. EDV (mm/s)**	28.52 ± 8.27	28.99 ± 6.55	28.98 ± 2.33	29.01 ± 5.62	29.21 ± 3.66
**VA. S/D**	4.21 ± 0.32	4.33 ± 0.55	4.45 ± 0.02	4.31 ± 0.25	4.55 ± 0.22
**VA. PI**	1.66 ± 0.02^b^	1.65 ± 0.02^b^	1.52 ± 0.02^ab^	1.51 ± 0.02^ab^	1.02 ± 0.02^a^
**VA. RI**	0.66 ± 0.02^b^	0.64 ± 0.02^b^	0.56 ± 0.02^ab^	0.54 ± 0.02^ab^	0.48 ± 0.02^a^

*SE* Standard error, *H* Horn, *ET* Endometrium thickness, *VA* Vaginal artery, *MUA* Middle uterine artery, *PSV* Peak point of velocity, *EDV* End point of velocity, *S/D* Systolic over diastolic ratio, *PI* Pulsatility Doppler index, and *RI*  Resistance Doppler index

Means with different superscripts ( a, b, ab) are significant different at *P* < 0.05

**Fig. 6:**
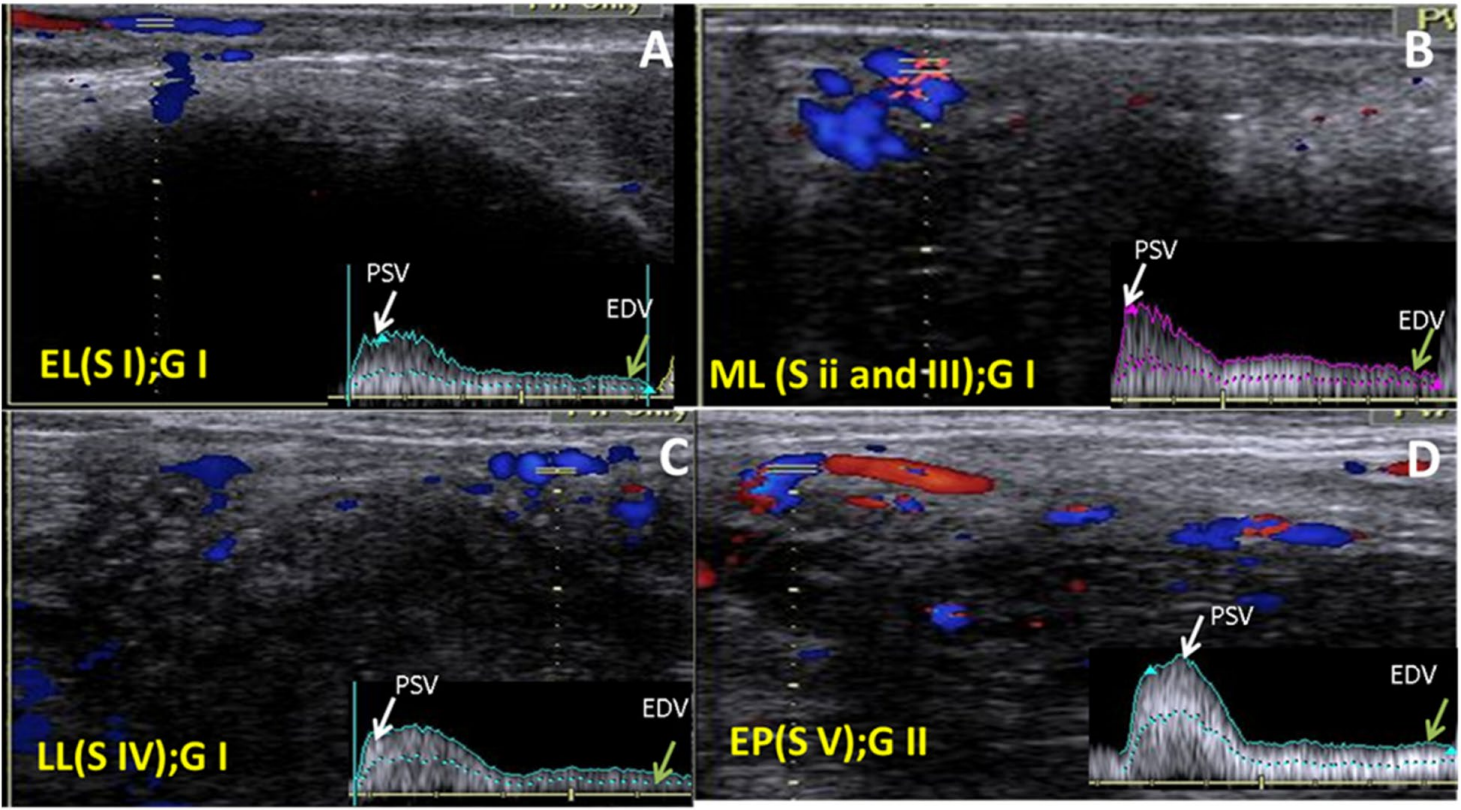
Ultrasonograms revealed the pulsed wave Doppler mode of the vaginal artery in buffalos in two groups. **A** colored picture showed the vaginal artery spectral graph with a white arrow at the peak point of velocity (PSV mm/sec) and green arrow at the end point of velocity (EDV/mm/sec) at the early luteal (Stage I). **B** Colored picture showed the vaginal artery spectral graph with PSV mm/sec and EDV/mm/sec at the mid luteal (Stage II and III). **C** Colored picture showed the vaginal artery spectral graph at the late luteal (Stage IV), and **D** Colored picture showed the vaginal artery spectral graph in the early pregnant (Stage V). EL = early luteal, ML = mid luteal, LL = late luteal, S = stage, and G = group

Pearson correlation coefficients were applied between vascularity index and blood flow parameters during early, mid, and late luteal stages. There was a positive Pearson correlation between vaginal artery and uterine artery hemodynamic parameters while a negative correlation was observed between Doppler indices and blood flow velocities in both arteries in early, mid, and late luteal stages in Egyptian domestic buffalos **(**Fig. 6A, B, and C). As VA PSV was positively correlated with VA EDV (*r *= 0.88, *P *= 0.02), MUA PSV (*r *= 0.87, *P *= 0.04), MUA EDV (*r *= 0.77, *P *= 0.02), while the same parameter was negatively correlated with VA. S/D (*r *= -0.77, *P *= 0.04), VA.PI (*r* = -0.89, *P *= 0.01), VA. RI (*r *= -0.97, *P *= 0.02), MUA. S/D (*r *= -0.94, *P* = 0.01), MUA. PI (*r *= -0.85, *P* = 0.04), and MUA. RI (*r *= -0.88, *P* = 0.04). In addition, both Doppler indices (PI and RI) were negatively corrected with the VA PSV (*r *= -0.68, *P* = 0.04), VA EDV (*r* = -0.74, *P *= 0.02), MUA PSV (*r *= -0.89, *P *= 0.01), and MUA EDV (*r *= -0.68, *P *= 0.01), while both parameters were positively correlated with VA S/D (*r *= 0.87, *P* = 0.01) and MUA S/D (*r *= 0.93, *P *= 0.02). There was a positive correlation between the vaginal artery and uterine artery hemodynamic parameters while a negative correlation was observed between Doppler indices and blood flow velocities in both arteries in early pregnant stage in Egyptian domestic buffalos (Fig. 6D). As VA PSV was positively correlated with VA EDV (*r *= 0.98, *P *= 0.01), MUA PSV (*r *= 0.67, *P *= 0.01), MUA EDV (*r *= 0.77, *P *= 0.02), while the same parameter was negatively correlated with VA. S/D (*r *= -0.87, *P* = 0.01), VA.PI (*r *= -0.84, *P* = 0.01), VA. RI (*r *= -0.91, *P* = 0.01), MUA. S/D (*r *= 0.58, *P *= 0.54), MUA. PI (*r *= -0.85, *P *= 0.04), and MUA. RI (*r *= -0.88, *P* = 0.04). In addition, both Doppler indices (PI and RI) were negatively corrected with the VA PSV (*r *= -0.68, *P *= 0.04), VA EDV (*r *= -0.72, *P *= 0.01), MUA PSV (*r *= -0.59, *P *= 0.04), and MUA EDV(*r *= -0.66, *P* = 0.05), while both parameters were positively correlated with VA S/D (*r *= 0.87, *P *= 0.01) and MUA S/D (*r *= 0.97, *P *= 0.03).

**Fig. 7:**
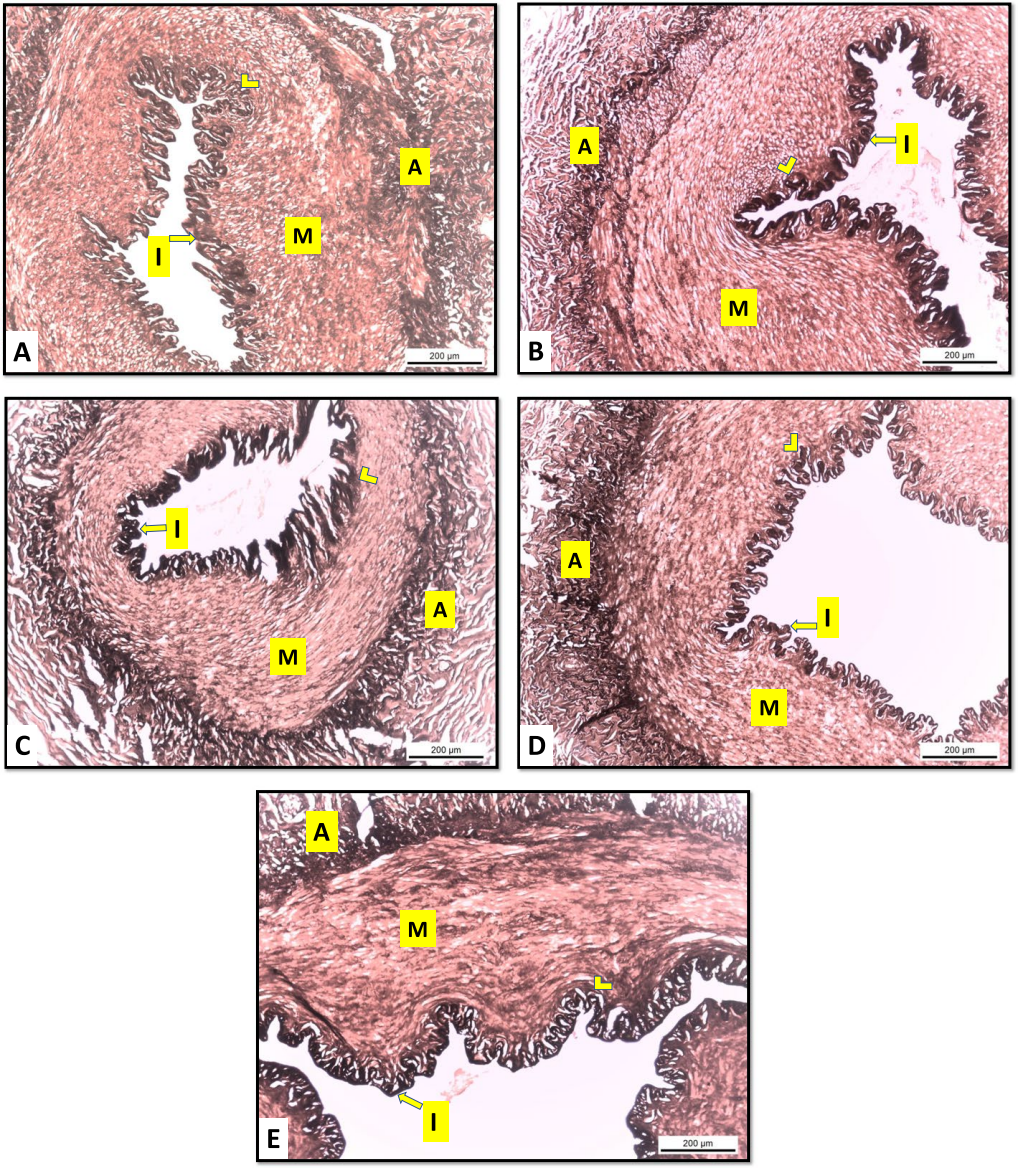
Cross section in the vaginal artery of Egyptian buffalo at different luteal stages A: early luteal, B: mid-luteal (stage II), C: mid-luteal (stage III), D: late luteal, E: pregnant, I: tunica intima, lamina elastica interna (arrowhead), M: tunica media, A: tunica adventitia. Orcein stain. X100

The histological examination of the vaginal artery of Egyptian buffalo at different luteal stages revealed that it was a muscular artery consisting of three tunics; tunica intima; thin endothelium rest on thin connective tissue layer, thick tunica media “tunica muscularis” composed of concentrically arranged smooth muscle fibers with few elastic fibers in between. The two tunics were separated by a wavy elastic membrane called lamina elastica interna and the last tunica was tunica adventitia which contained collagen and more elastic fibers (Fig. 7).

Histomorphometrical analysis of the data of the vaginal artery revealed that the mean values of the luminal diameter gradually increased with significant differences (590.99±21.81 and 868.53±30.13 μm) in the late luteal and pregnant stages than other stages (453.86±16.3, 444.14±43.29, and 403.57±21.90 μm) early, mid-luteal II, and III stages respectively. On the other hand, the thickness of the tunica muscularis was significantly increased in the pregnant stage (616.86±36.61 μm) than in other stages (235.13± 9.37, 330.83±14.21, 293.08±24.69, and 385.23±9.24 μm) early, mid-luteal II, III, and late luteal stages respectively (Table 4).

**Table 4:** Histomorphometrical analysis of luminal diameter and tunica muscularis thickness of the vaginal artery of Egyptian buffalo at different luteal and pregnant stages

Means ± SE	Stages				
	Early luteal	Mid luteal “stage II”	Mid luteal “stage III”	Late luteal	Pregnant
**Luminal diameter (**μm**)**	453.86 ± 16.3^a^	444.14 ± 43.29^ab^	403.57 ± 21.90^abc^	590.99 ± 21.81^d^	868.53 ± 30.13^e^
**Tunica muscularis thickness (**μm**)**	235.13 ± 9.37^a^	330.83 ± 14.21^b^	293.08 ± 24.69^abc^	385.23 ± 9.24^bd^	616.86 ± 36.61^e^

Means with different superscripts in the same row indicate significant difference *P*<0.05

^ab^means not significant with the early luteal phase but significant with other groups

^abc^means not significant with early and mid-luteal phase II but significant with other groups

^bd^means not significant with mid-luteal phase II but significant with other groups

## Discussion

The previous studies on the PVU of Egyptian buffalo are limited [30]. The present results may contribute to future studies when these animals are taken under protection and propagated by artificial insemination that differed and affected when inserted catheter into normal appearance of PVU than abnormal one .The PVU considered as the protrusion of the external uterine orifice into the vagina that formed the shape of cervix uteri which was found to be rose-shaped in sheep [4], in wild goats [6]. as that observed in our study in the mid-luteal (stage III), in Egyptian buffalo. Moreover, papilla shaped in lambs [4] as appeared in early stage in our findings. The width of the PVU of Egyptian buffalo in this research was 25mm in the early luteal stage, 30mm in the middle luteal stage II, 40mm in mid-luteal stage III, 55mm in the late luteal stage, and 40mm in pregnant stage, none of the available literatures discussed this point previously in buffalo. On the other hand, the width of the PVU was measured in different species of goats as 14.1 mm in Gaddi goats [31], 10.7 mm in red Sokoto goats [32], 17.55 mm in Black Bengal goats [33], and 13.39 mm, in wild goats [6].

The characteristics of cervical mucus were modified according to the effect of ovarian hormones secreted during estrus that act on the mechanical barrier to sperm motility, and facilitate the fertilization rate [34, 35]. Regarding our observation, the present study showed few clear watery vaginal secretions in the mid-luteal (stage III), and late luteal stages that are similar to the finding reported by [36, 37]. Indian buffaloes showed a viscous (thick mucoid) secretion appeared in a pregnant stage that is in agreement with cows and heifers [38, 39].

Endocervical secretory cells form the cervical mucous. The quantity and quality of it are influenced by the gonadal hormone levels during the estrous cycle [40]. Because of the changes that take place in the cervical secretions during the estrous cycle and during pregnancy, they are interesting. The mucous content of the buffalo's PVU epithelium contains various mucopolysaccharides, which could be stained by AB and PAS. It stained bright magenta with PAS due to presence of neutral mucin granules in the apical cytoplasm of the epithelial cells and the acidic mucins-stained intense blue with AB.

The current study illustrated a significant difference in the area % of AB and PAS-positive granules in the epithelial cells of PVU mucosa which were increased in the pregnant luteal stage than in other luteal stages. Similar to what has been seen for the bovine cervical mucosa [41], the percentage of cervical epithelial cells with PAS-positive granules was higher in pregnant females and the luteal phase compared to the follicular phase [42]. Blood vessels in genital organs dilate when estrogen levels are high, and cervical and vaginal glands secrete mucous [43].

Our results asserted that the vaginal artery originated from the internal iliac artery as that mentioned by [27, 44]. While arose together with middle uterine artery in a common trunk from the umbilical artery in case of she-camel [7]. The vaginal artery in buffalo was divided into two main branches; large cranial branch and small caudal one , that was inversely in diameter in she-camel as the cranial branch was the smaller one that confirmed by [7].

In this study, alterations in the VA blood flow in buffalos were documented for the first time regarding to histological and anatomical references during the luteal and early pregnant stages. In ruminants such as sheep and buffalos, MUA and vaginal artery alterations were reported [44–46]. with an increase in cell volume. These alterations affecting the protein content by elevation in concentrations (actin and myosin) lead to cellular hypertrophy in early pregnant animals with elevation of the vascular wall blood flow and reduction of the vascular resistance to the blood flow (due to placentation) with association to pulsatility Doppler index [47–49].

The positive correlation that was detected in the current study between Doppler indices in the MUA and VA was in accordance with many literatures reported the positive relationship between both arterial hemodynamic alterations as the uterine artery increase within 2-3 folds in the early pregnant stage compared to its level in the luteal stages in human [50, 51], and animals [52, 53] and the vaginal artery behave the similar pattern of change as it is considered a branch from the MUA and sometimes called caudal uterine arterial branch [54].

Although, the histological examination of the vaginal artery of Egyptian buffalo at different luteal stages demonstrated a significant increase in the luminal diameter and the thickness of tunica muscularis in the pregnant stage than other luteal stages. These findings were in line with Abouelela et al. [55]. who recorded significantly increased luteal, ovarian, and uterine arteries lumen diameter mean values in pregnant buffalos (309 ± 0.84, 840.2 ± 0.74, and 961 ± 0.07 μm) than in non-pregnant buffalos (291.2 ± 0.80, 342 ± 0.45, and 854 ± 0.12 μm). In contrast, the luteal and uterine arteries of non-pregnant buffalos had mean values of the tunica media's thickness (206 ± 0.09 and 556 ± 0.11 μm) were significantly increased than those of pregnant buffalos (134 ± 0.11 and 440 ± 0.1 μm), according to the same authors. A significant elevation in the arterial (uterine and vaginal) wall thickness with a marked thickening in the histological vascular wall was reported and in accordance with Osol; Osol and Cipolla [51, 56]. Some reports determined the same elevation especially in the gestational stage [57, 58]. The increased luminal diameter was attributed to hypertrophy “increased in the cell volume” in addition to hyperplasia “cell proliferation”. The majority of the arterial wall becomes filled with the tunica muscularis. Stress occurred by the pregnancy event was the main cause of the arterial wall alterations [51]. One of the limitations of this work is lack studying of the ovaries and the whole reproductive organs at the studied stages to give a complete overview at this aspect. In addition, comparison between different species was also missed at our research. Thus, we recommend researchers to resume in this subject to fill this gap.

## Conclusion

It was concluded that there was a significant alterations in histological features of the cervical PVU at different physiological stages (luteal and early pregnant) in buffalos in relation to the MUA and VA hemodynamic pattern and that hypotheses can be established regarding the female cyclicity that affected by both arteries hemodynamics change. In addition, knowing the visual structure of PVU could help in designing the effective instrumentation and techniques for trans cervical passage of semen during artificial insemination in this species.

## Data Availability

The authors confirm that the data used to support the findings of this study are available within the article .

## Abbreviations

PVU: Portio vaginalis uteri
VA: Vaginal artery
ET: Endometrial thickness
PI: Pulsatility index
S/D: Systolic over diastolic ratio
MUA: Middle uterine artery diameter
PSV: Peak points of velocities
EDV: End points of velocities
RI: Resistance index
H: Horn diameter
